# Draft Genome Sequence of an Escherichia coli Sequence Type 155 Strain Isolated from Sewage in Kerala, India

**DOI:** 10.1128/MRA.01707-18

**Published:** 2019-07-03

**Authors:** Amrita Salim, Pradeesh Babu, Keerthi Mohan, Manju Moorthy, Devika Raj, Swathy Kallampillil Thirumeni, Sagarbabu Suresh, Ajith Madhavan, Bipin G. Nair, Sujay Chattopadhyay, Sanjay Pal

**Affiliations:** a School of Biotechnology, Amrita Vishwa Vidyapeetham, Kollam, Kerala, India; University of Maryland School of Medicine

## Abstract

We report the draft genome sequence of Escherichia coli ASBT-1, a representative of E. coli sequence type 155 (ST155), obtained from India. Considering the known wide variety of pathogenic and antibiotic resistance potentials, this strain should be of great interest for detailed comparative genomic analysis.

## ANNOUNCEMENT

Escherichia coli sequence type 155 (ST155) represents important strains responsible for zoonotic transmission of extended-spectrum β-lactamase genes to humans (1–8). We announce an assembled draft genome of an E. coli ST155 strain obtained from wastewater in Kerala, India, and explore the diversity of different antibiotic resistance profiles in the region.

The organism was isolated from sewage in eosin-methylene blue agar, biochemically characterized as E. coli, and confirmed by 16S rRNA gene ribotyping (9). Genomic DNA was extracted using the phenol-chloroform method (10). The paired-end sequencing library was prepared using the TruSeq Nano DNA library prep kit. The Illumina HiSeq platform was used for sequencing the paired-end library, with a read length of 2 × 150 bp and coverage of 850×. Both quantity and quality checks of the amplified library were performed in a Bioanalyzer 2100 (Agilent Technologies) using a high-sensitivity DNA chip per the manufacturer’s instructions. The reads generated were filtered using Trimmomatic (v0.30) with a quality value (QV) of >20, and adapters were also removed. Subsequently, the high-quality (4.15 Gb) data were used for assembly. *De novo* assembly of paired-end reads was performed using Velvet v1.2.10. The total number of reads was 28,029,838. The details of the assembled genome are listed in Table 1. An NCBI genome annotation tool was used to annotate the genome and detected a total of 4,393 protein-coding genes with an average size of 945 bp. This genome was found to harbor 75 tRNA and 8 rRNA genes, as predicted by tRNAscan-SE v2.0 (11) and DFAST v1.0.1 (12), respectively. A total of 5 intact prophage regions were identified using the PHAST tool (13) (last accessed date, 18 September 2018). Two CRISPR-Cas sequences were detected, one by CRISPRFinder (14) (last accessed date, 18 September 2018) and one by Prokka v1.12 (15), respectively. Altogether, as an Indian representative of an E. coli ST155 clone, ASBT-1 warrants additional in-depth research on its genomic features, pathotype, and antibiotic resistance profile.

**TABLE 1:** Summary of the genome sequence of strain ASBT-1

Assembly or annotation element	Data
Genome size (bp)	4,696,000
No. of contigs	54
No. of scaffolds	50
Scaffold *N*_50_ (bp)	383,050
Avg scaffold length (bp)	93,920
GC content (%)	50.81
No. of protein-coding genes	4,393
Coding ratio (%)	88.1
Avg protein length (amino acids)	313.9
No. of tRNAs	75
No. of rRNAs	8
No. of CRISPRs	2
No. of intact prophage regions	5

### Data availability.

This whole-genome shotgun project has been deposited at DDBJ/ENA/GenBank under the accession number RWJY00000000 (BioProject number PRJNA509104). The version described in this paper is RWJY01000000, with SRA accession number SRR8480428.
